# Characterization data of reference industrial polycarboxylate superplasticizers used within Priority Program DFG SPP 2005 “Opus Fluidum Futurum - Rheology of reactive, multiscale, multiphase construction materials”

**DOI:** 10.1016/j.dib.2020.106026

**Published:** 2020-07-15

**Authors:** L. Lei, C. Chomyn, M. Schmid, J. Plank

**Affiliations:** Technische Universität München, Chair for Construction Chemistry, 85747 Garching, Lichtenbergstraße 4, Germany

**Keywords:** Polycarboxylate, Characterization, Molecular characteristics, Dispersing property, Fluidity, DFG SPP 2005

## Abstract

Two industrial polycarboxylate superplasticizer samples have been selected to be used within the Priority Program 2005 of the German Research Foundation (DFG SPP 2005). The PCE polymers were characterized via Size Exclusion Chromatography (SEC) to determine their molar masses (*M_w_, M_n_*), the polydispersity index (PDI) and the conversion rate which indicates the incorporation of the macromonomer into the polymer. The anionic charge amount of the PCE samples was assessed via charge titration employing a cationic polymer. Furthermore, the cement dispersing properties of the PCE polymers were captured via 'mini slump' tests so as to assess their ability to fluidize CEM I 42.5 R and CEM III/A 42.5 N samples, respectively. Also, interaction between the PCEs and the surface of the cements was investigated via adsorption and zeta potential measurements of aqueous cement suspensions. The results shall be used for the ongoing research within the Priority Program.

    Specifications Table

**Table:** 

Subject	Polymers and Plastics
Specific subject area	Admixture for concrete; Dispersant; Polyethylene glycol derivatives
Type of data	Table; Graph; Figure
How data were acquired	Size exclusion chromatography; PCD 03 pH particle charge detector; High TOC II Instrument; DIN EN 1015; DT 1200 Electroacoustic Spectrometer; Infrared balance; pH meter
Data format	Raw; Analyzed
Parameters for data collection	Solid content; Density; Anionic charge density; Molar masses (*M_w_, M_n_*); polydispersity index; Conversion; Spread flow; Adsorbed amount; Zeta potential
Description of data collection	The data were obtained at the Chair for Construction Chemistry, Prof. Dr. J. Plank, TU München.
Data source location	Technische Universität München, Chair for Construction Chemistry, 85747 Garching, Lichtenbergstraße 4, Germany
Data accessibility	Repository name: mediaTUM Data identification number: https://doi.org/10.14459/2020mp1547135 Direct URL to data: https://mediatum.ub.tum.de/1547135

## Value of the Data

• This dataset is the detailed record of physical, chemical and application related properties of the polycarboxylate superplasticizers used in the Priority Program 2005 of the German Research Foundation (DFG SPP 2005).
• All the research groups involved in the DFG SPP 2005 Priority Program and other related researchers can use these data for their further study.
• The research partners within the SPP 2005 are using the polymers described here as dispersing agents for cement, silica and other colloidal particles as an essential component in their fluid systems for which they produce rheological data e.g. relating to shear-rate dependent flow behavior.
• The data on the polymers such as chemical structure, *M_w_*, anionic charge, purity etc. will enable the researchers to understand their experimental results better and to gain more insights into surface interactions, inter-particle forces etc.
• The data can help the researchers within the SPP framework to understand their experimental data better and to gain more insight into the ongoing project.

## 1 Data Description

This Data in Brief article provides the detailed information regarding the analysis of the physical properties, chemical compositions and molecular characteristics of two polycarboxylate superplasticizers provided by BASF company. In addition, the dispersing effectiveness of these two polycarboxylate superplasticizers in CEM I 42.5 R and CEM III/A 42.5 N cement samples was assessed via ‘mini slump’ tests and their interaction with those cements was characterized via adsorption and zeta potential measurements.

### 1.1 Characterization data of physical properties

Table 1 reports the solid content, density and pH value of the BASF PCE superplasticizers as measured.

**Table 1:** Solid content, density and pH value of BASF PCE superplasticizers.

Product	Solid content [wt. %]	Density [kg/L]	pH
VP2018/13.1 (precast)	19.5	1.04	5.2
VP2018/14.1 (ready-mix)	22.6	1.06	6.0

### 1.2 Chemical compositions and molecular properties

**Fig. 1:**
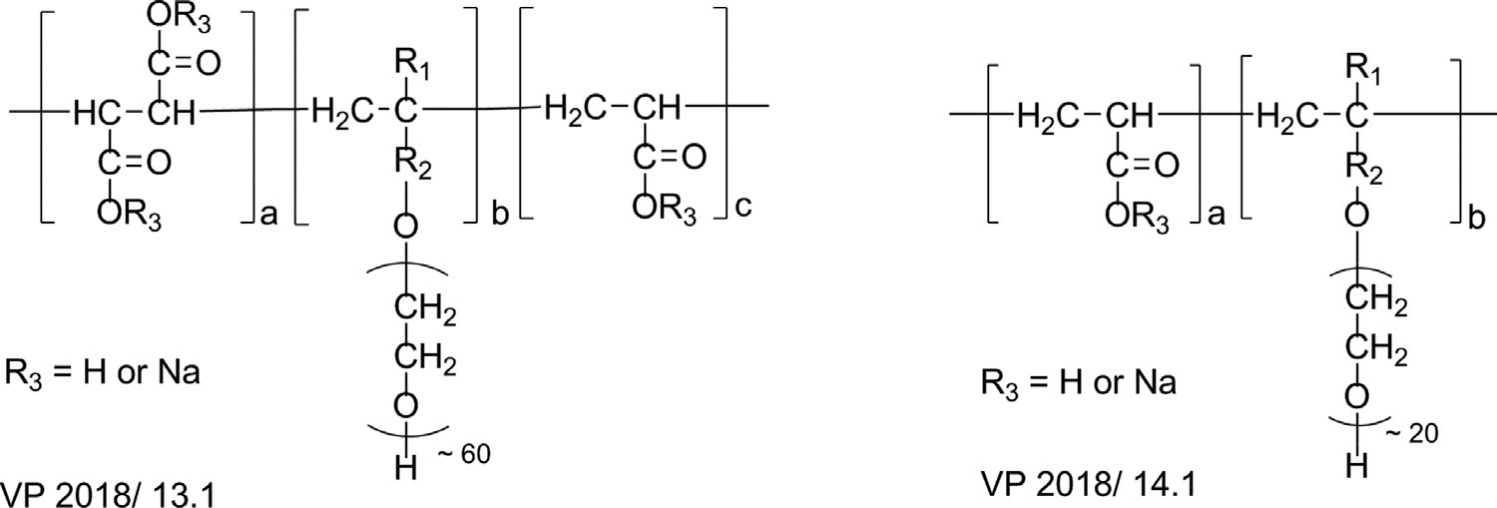
Chemical structures of the industrial BASF PCE superplasticizer samples

Fig. 1 displays the chemical structures of the BASF PCE superplasticizers.

VP 2018/13.1 represents an ether based PCE. The number of ethylene oxide units in the side chain is about 60.

VP 2018/14.1 represents an ether based PCE. The number of ethylene oxide units in the side chain is about 20.

**Fig. 2:**
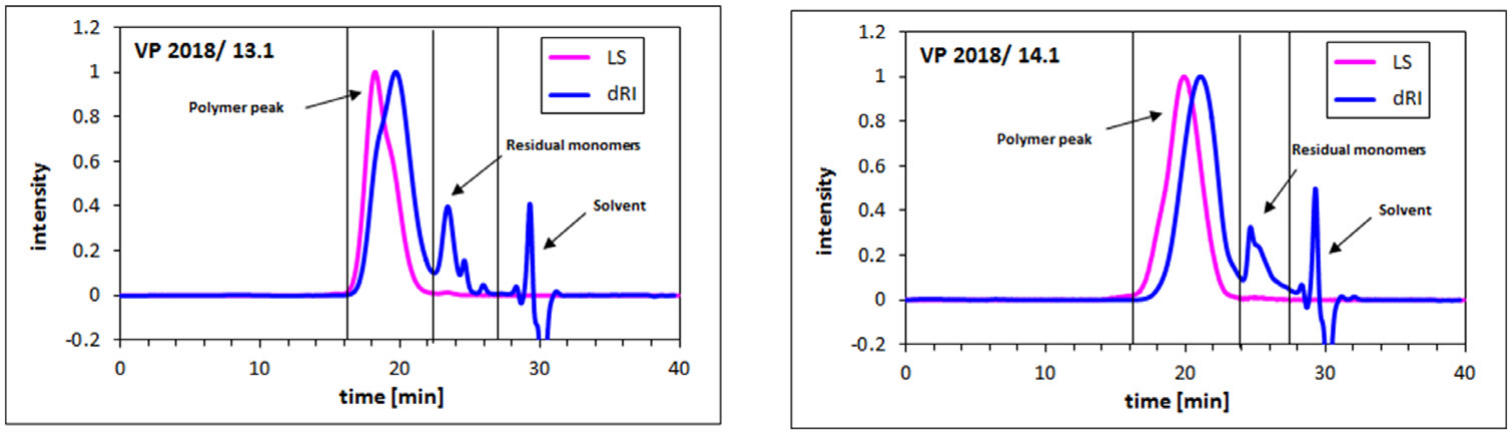
SEC spectra of the BASF PCE superplasticizers; eluent: 0.1 M NaNO_3_.

The molecular properties of the two BASF PCE superplasticizers were obtained via Size Exclusion Chromatography (SEC). In Fig. 2, the SEC spectra of two samples are displayed. Molar masses (*M_w_, M_n_*), polydispersity index (PDI) and conversion of the macromonomer are listed in Table 2.

**Table 2:** Molar masses, PDI and macromonomer conversion for BASF PCE superplasticizers.

Property	VP 2018/13.1	VP 2018/14.1
*M_w_* [g/mol]	98,680	28,090
*M_n_* [g/mol]	47,220	11,770
PDI	2.09	2.39
Macromonomer Conversion [%]	81.7	84.4

### 1.3 Characterization data of anionic charge properties

**Fig. 3:**
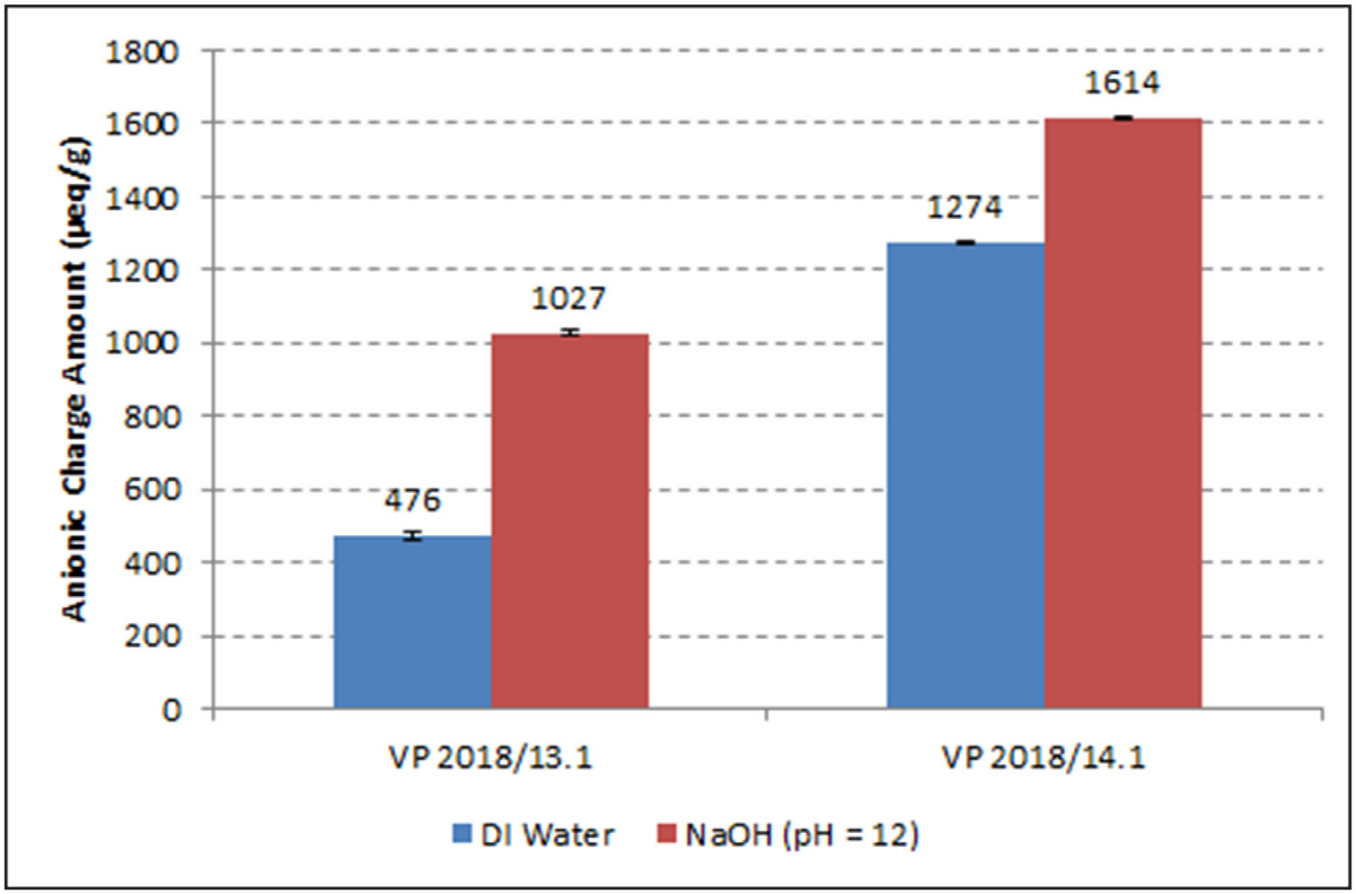
Anionic charge amounts of the BASF PCE superplasticizer samples.

Anionic charge amounts of the PCE superplasticizer samples were determined in DI water and in 0.01M aqueous NaOH solution (pH = 12) via polyelectrolyte titration using cationic polydiallyl dimethyl ammonium chloride (polyDADMAC) as a titrator. The tests were performed utilizing a PCD 03 pH particle charge detector (Mütek Analytic, Herrsching, Germany). The results are shown in Table 3 and Fig. 3, respectively.

**Table 3:** Anionic charge amount of BASF PCE superplasticizers.

Product	Anionic charge amount [μeq/g]	
	DI water	0.01 M NaOH, pH = 12
VP 2018/13.1	476	1027
VP 2018/14.1	1274	1614

### 1.4 Dosage - dependent dispersing effect in CEM I 42.5 R

**Fig. 4:**
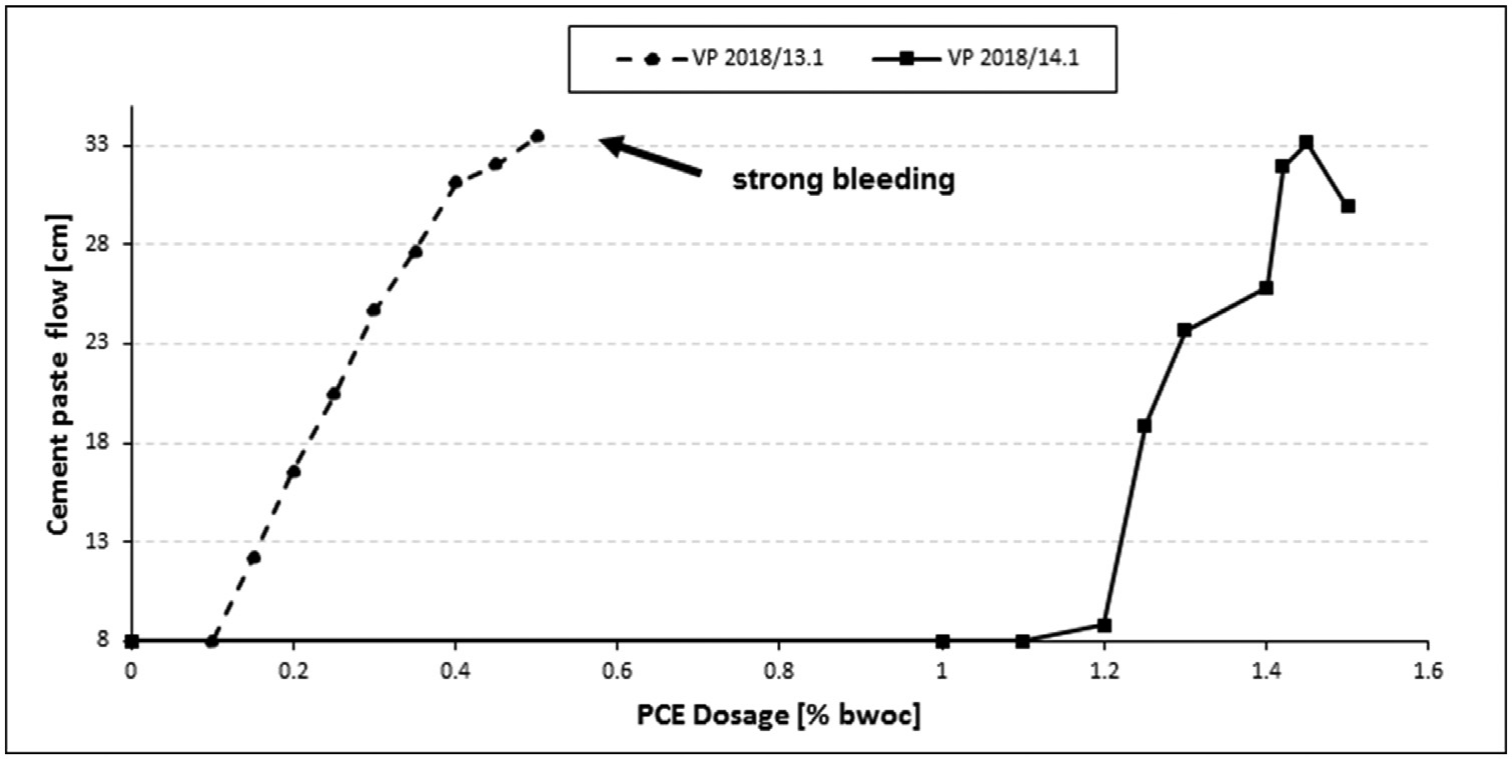
Dosage - dependent cement paste flow of BASF PCE samples in CEM I 42.5 R (w/c = 0.3).

The cement dispersing effect was investigated via a modified “mini slump” test according to DIN EN 1015 [1] at 20°C and 40 % rel. humidity. A water-to-cement ratio of 0.30 for CEM I 42.5 R and of 0.31 for CEM III/A 42.5 N was used according to the water demand determined for both cements [2]. The plot of cement paste flow against PCE dosage is displayed in Fig. 4.

At the water-to-cement ratio of 0.3, the dosage required for VP2018/14.1 to achieve maximum fluidity is extremely high, ∼ 1.45 % by weight of cement (bwoc).

**Fig. 5:**
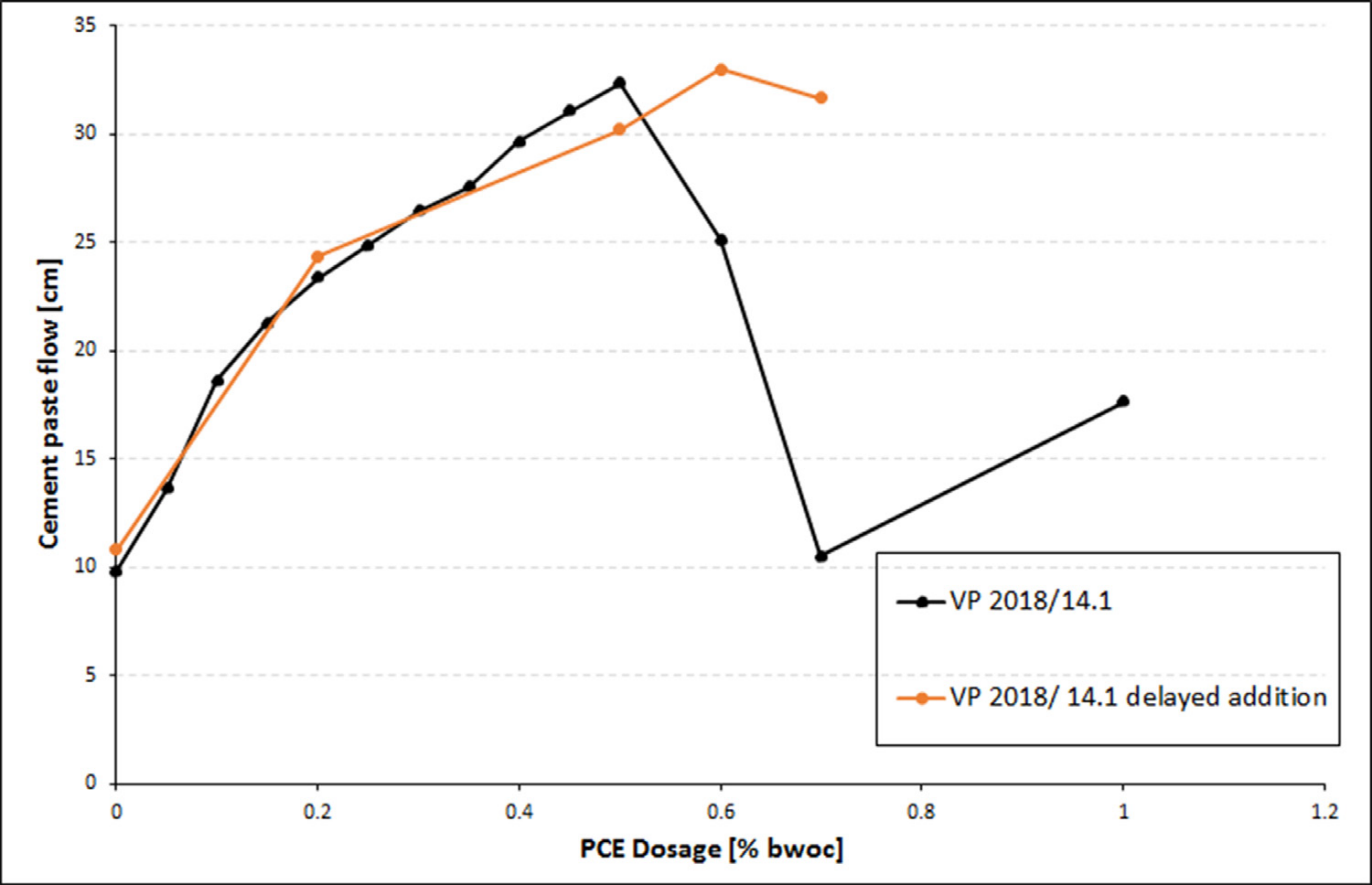
Dosage - dependent cement paste flow of sample VP2018/14.1 in CEM I 42.5 R (w/c = 0.4).

Furthermore, the dosage - dependent dispersing behaviour of this PCE sample VP 2018/14.1 was assessed at a higher w/c ratio of 0.4 (instead of 0.3 in the previous test). The results are displayed in Fig. 5. There, also the dispersing capacity of VP 2018/ 14.1 sample is shown when the liquid PCE is dosed to the freshly prepared cement paste (= delayed addition) instead of being dissolved in the mixing water as was done in the previous tests.

### 1.5 Dosage - dependent dispersing effect in CEM III/A 42.5 N

**Fig. 6:**
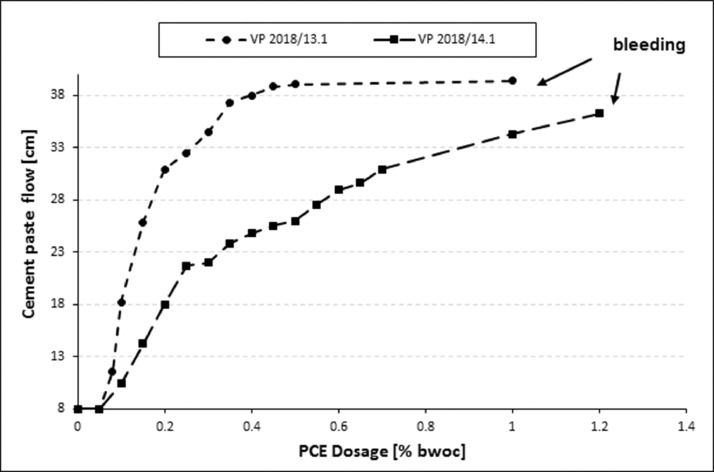
Dosage - dependent cement paste flow of the BASF PCE samples in CEM III/A 42.5 (**w/c = 0.31**).

The results for both PCE samples relating to this cement are displayed in Fig. 6**.** Tests were performed according to DIN EN 1015 [1], with the PCE being pre-dissolved in the mixing water.

### 1.6 Slump Retention Behaviour in CEM I 42.5 R

**Fig. 7:**
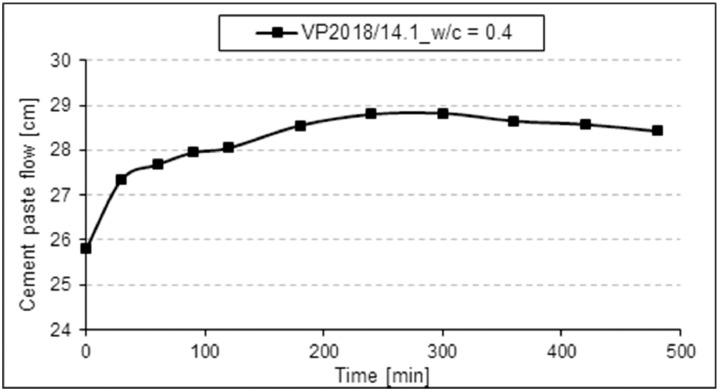
Slump retention of PCE sample VP 2018/14.1 in a paste prepared from CEM I 42.5 R.

The paste spread flow over time of sample VP 2018/14.1 (ready-mix type PCE) was recorded and is presented in Fig. 7. A w/c value of 0.4 was used to adjust a paste flow of 26 ± 0.5 cm as starting flow value. The PCE dosage to obtain the 26 cm flow was 0.30 % bwoc (see Fig. 5). Tests were performed according to DIN EN 1015 [1], with the PCE being pre-dissolved in the mixing water as above.

### 1.7 Adsorption of PCE samples on CEM I 42.5 R and CEM III/A 42.5 N

*Langmuir* adsorption isotherms were developed for the PCEs on both cement samples by adding increasing dosages of the PCEs to the cement pastes which were then centrifuged and the amount of non-adsorbed PCE present in the supernatant was determined via total organic carbon (TOC) measurement. The amounts of the PCEs adsorbed were then calculated according to the depletion method.

**Fig. 8:**
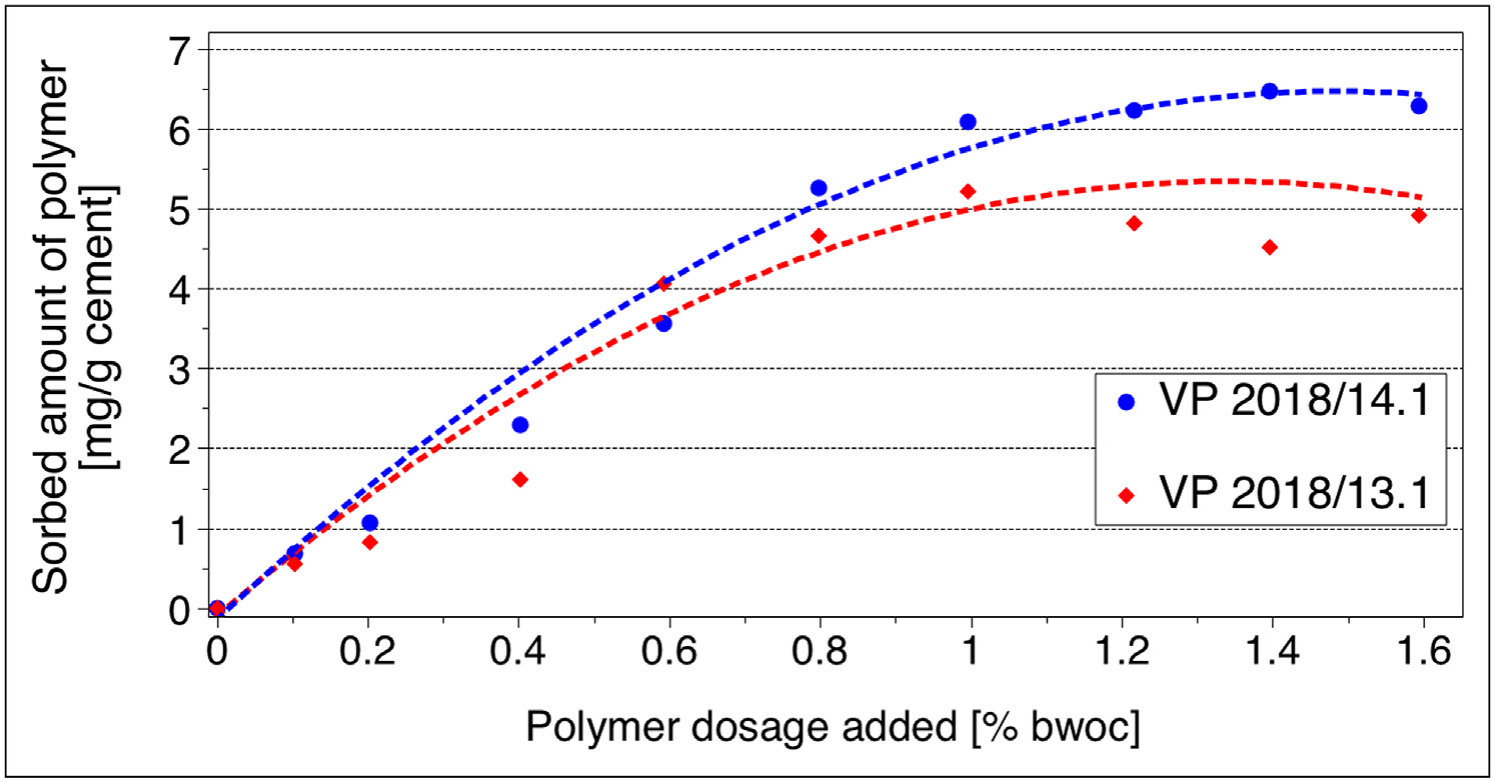
Adsorption isotherms for VP 2018/13.1 and VP 2018/14.1 on **CEM I 42.5 R**; cement paste, w/c ratio = 0.5.

The adsorption isotherms for VP 2018/13.1 and VP 2018/14.1 on CEM I 42.5 R are displayed in Fig. 8**.**

**Fig. 9:**
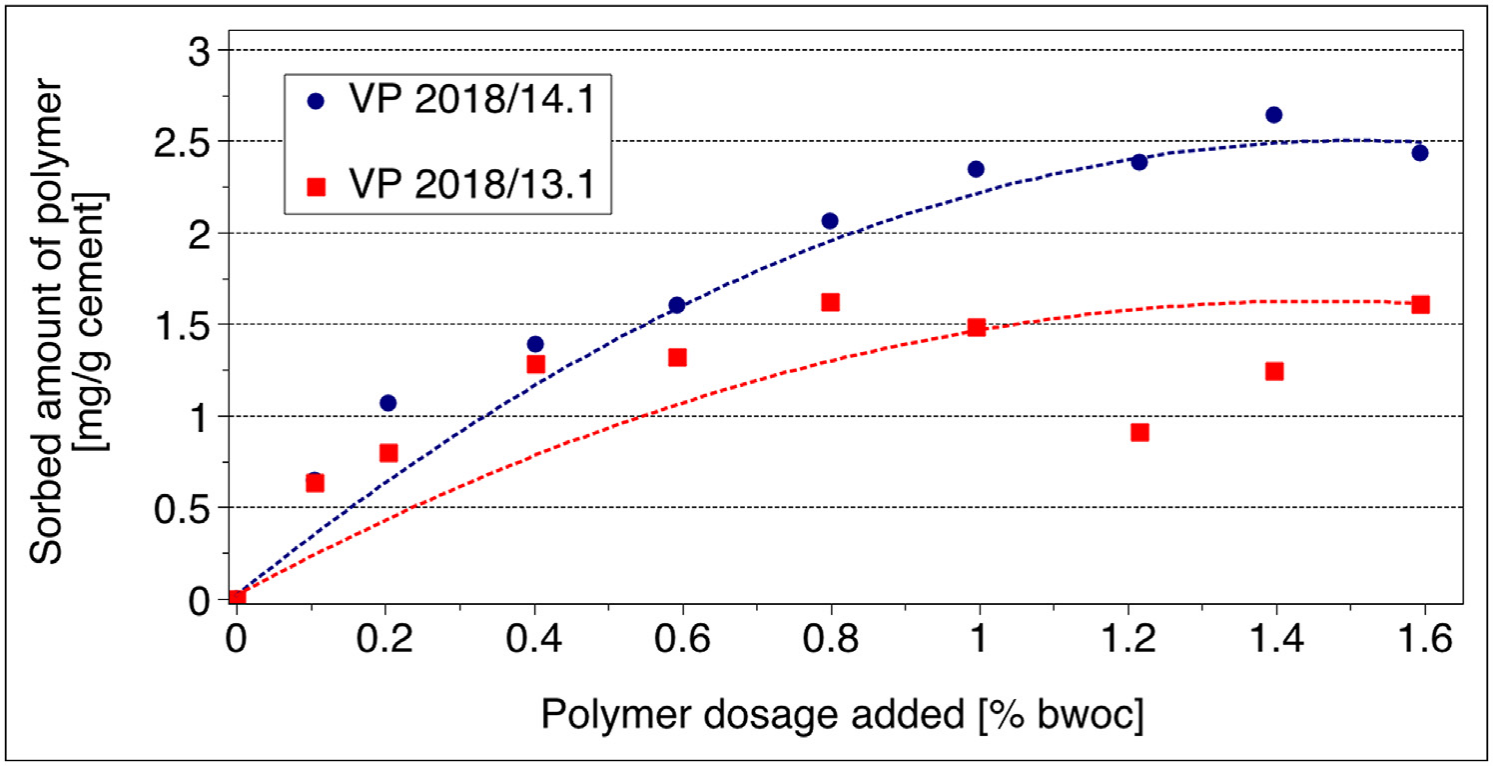
Adsorption isotherms for VP 2018/13.1 and VP 2018/14.1 on **CEM III/A 42.5 N**; cement paste, w/c ratio = 0.5.

The adsorption isotherms for VP 2018/13.1 and VP 2018/14.1 on CEM III/A 42.5 N are displayed in Fig. 9**.**

### 1.8 Zeta potential of cement suspensions treated with PCEs

Interaction between the PCEs and the surface of the cements was characterized via zeta potential measurements of aqueous cement suspensions whereby the PCE polymers were gradually dosed in.

The electro-kinetic properties of the slurries were measured using a Model DT-1200 electroacoustic Spectrometer (Dispersion Technology, Inc., Bedford Hills, NY, USA). To develop the zeta potential as function of PCE dosage, aqueous solutions of the PCE samples (concentration 10 wt. %) were stepwise titrated to the cements suspended in water (w/c = 0.5). The zeta potentials of the slurry were recorded as a function of PCE addition.

**Fig. 10:**
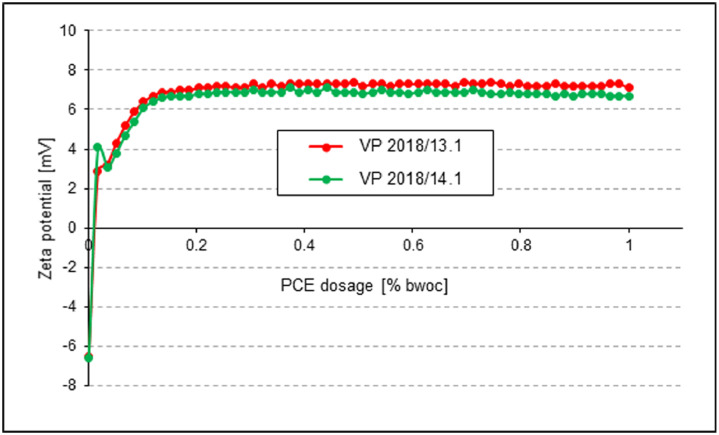
Dosage-dependent zeta potentials of pastes prepared from **CEM I 42.5 R**, treated with VP 2018/13.1 or VP 2018/14.1 (w/c ratio = 0.5).

**Fig. 11:**
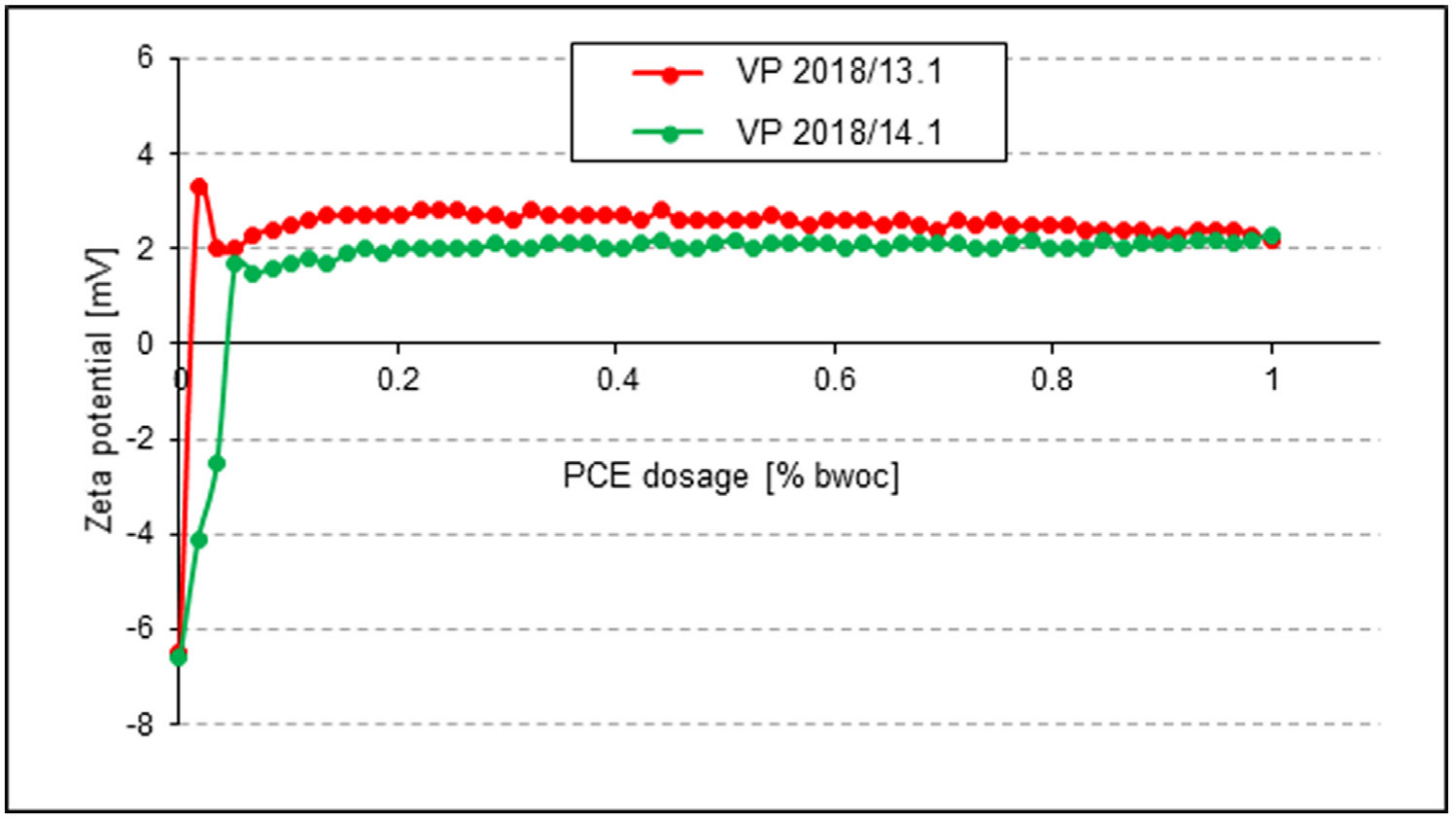
Dosage-dependent zeta potentials of pastes prepared from **CEM III/A 42.5 N**, treated with VP 2018/13.1 or VP 2018/14.1 (w/c ratio = 0.5).

The results of the titration experiments are shown in Fig. 10 for CEM I 42.5 R slurries and in Fig. 11 for CEM III/A 42.5 N slurries, respectively.

## 2 Experimental Design, Materials, and Methods

**Molecular properties**: Molar masses (*M_w_* and *M_n_*) of the PCE samples and the conversion of the macromonomers were determined *via* size exclusion chromatography (SEC) which is also known as gel permeation chromatography (GPC). Signals were collected from light scattering (red curve in Fig. 2, molar mass dependent) and refractive index (blue curve in Fig. 2, concentration dependent). The polydispersity index (PDI) which is derived from the quotient *M_w_*/*M_n_* indicates how broad the molecular weight distribution is, i. e. the larger the PDI value, the broader the molecular weight distribution of the sample. For this study, a Waters Alliance 2695 separation instrument (Waters, Eschborn, Germany) equipped with a three-angle static light scattering detector (“mini Dawn” from Wyatt Technology Corp., Santa Barbara, CA) was employed. Separation of the polymer fractions was achieved by using three Ultrahydrogel^TM^ (120, 250, and 500) columns and a Ultrahydrogel^TM^ guard column (Waters, Eschborn, Germany). The eluent was composed of 0.1 M NaNO_3_ and 0.1 g/L NaN_3_ adjusted to pH = 12. The value of *d_n_*/*d_c_* applied to calculate the molar masses of the PCEs was 0.135 mL/g (value for polyethylene oxide) [3].

**The anionic charge amounts** of the PCE samples were determined with the help of a particle charge detector PCD 03 pH (Mütek Analytic, Herrsching, Germany). In a typical measurement, 0.2 g/L of the polymers were dissolved in DI water or 0.01 M NaOH solution (pH = 12) and titrated against a 0.34 g/L aqueous solution of polydiallyl dimethyl ammonium chloride (polyDADMAC) until charge neutralization (zero potential) was reached. From the amount of polyDADMAC consumed to reach zero potential, the amount of negative charge per gram of polymer (= the anionic charge amount) was calculated [4].

**The dispersing effectiveness and slump retaining properties** of the PCE polymers were investigated via “mini slump” test according to DIN EN 1015. In experiment, the mixing water and the polymer solution were placed in a porcelain cup. The mixture was stirred until a clear solution was obtained. Then, 300 g of cement were added within one minute to the cup. After one minute of soaking time, the cement paste was stirred manually for two minutes and immediately filled to the brim of a Vicat cone (height 40 mm, top diameter 70 mm, bottom diameter 80 mm) which was placed on a glass plate. The cone was removed vertically and the resulting paste spread was measured twice, with the second measurement being perpendicular to the first. The averaged values were reported as final spread value. As for the slump retention tests, after the initial spread flow measurement the cement paste was stored in the porcelain cup covered with a wet towel and tested for fluidity at 30, 60, 90, 120, 180, 240, 300, 360, 420 and 480 minutes after initial preparation.

**Adsorption**: The amounts of PCEs adsorbed on the cements were determined by analysing the total organic carbon (TOC) content in the supernatant after contact between cement and PCE. In a typical experiment, 30 g of cement were added to 15 mL DI water (w/c = 0.5) holding different dosages of the superplasticizers VP 2018/13.1 or VP 2018/14.1. The pastes were filled into 50 mL centrifuge tubes, shaken for two minutes at 2,400 rpm in a wobbler (VWR International, Darmstadt/Germany) and centrifuged for ten minutes at 8,500 rpm in a centrifuge (Heraeus International, Osterode/Germany). For quantification of the total organic carbon content in the supernatant, a LiquiTOC-II apparatus from Elementar (Hanau/Germany) was used. Centrifugates were diluted with 0.1 N HCl to remove inorganic carbonates and to prevent the dissolution of carbon dioxide in the alkaline solution. The adsorbed amounts of the PCE samples were calculated by subtracting the concentration of PCE found in the centrifugate from the initial PCE concentration existing prior to contact with cement.

**Zeta potential:** The electro-kinetic properties of the cement slurries were obtained via zeta potential measurements. In experiment, 300 g of cement were added within one minute to 150 g of DI water (w/c ratio = 0.5) placed in a porcelain cup. The mixture was allowed to soak for one minute before it was stirred manually for two minutes with a spoon. For zeta potential measurement, the paste was poured into a glass container, then the zeta potential electrode, the titrator, the temperature probe and the pH meter were inserted into the paste and the mixture was stirred continuously at 200 rpm at RT while the zeta potential measurements were taken.

## CRediT authorship contribution statement

**L. Lei:** Conceptualization, Investigation, Supervision, Writing - review & editing. **C. Chomyn:** Investigation, Methodology, Data curation, Validation. **M. Schmid:** Writing - original draft. **J. Plank:** Resources, Supervision.

## Declaration of Competing Interest

The authors declare that they have no known competing financial interests or personal relationships that could have appeared to influence the work reported in this paper

## Appendix Supplementary materials

Image, application 1
